# Seizures following Intoxication with a Common Antituberculosis Drug

**DOI:** 10.1155/2019/8972574

**Published:** 2019-03-05

**Authors:** Adnane Lahlou, Saïd Benlamkaddem, Mohamed Adnane Berdai, Mustapha Harandou

**Affiliations:** Obstetric and Pediatric Intensive Care Unit, Hassan II Academic Hospital, Fez, Morocco

## Abstract

Isoniazid is an antimycobacterial agent commonly prescribed in most tuberculosis chemotherapy regimens. Its side effects are widely known including mainly liver toxicity and peripheral neuropathy. The toxic effects of isoniazid are dose-related. Toxic doses are estimated at 35–40 mg/kg and fatal doses at 150 mg/kg. Treatment consists primarily of basic life support, antiepileptic drugs, and pyridoxine. The case is of one of the children with intentional isoniazid poisoning, the drug belonging to an uncle currently under antituberculosis treatment, resulting in tonic-clonic seizures. During her hospitalization in the paediatric intensive care unit, her neurological status as well as her lab values was closely monitored, and despite initial deterioration, the patient was discharged from critical care a week after full recovery.

## 1. Introduction

We report the case of a previously healthy 15-year-old female child (height: 162 cm, weight: 45 kg), admitted to our intensive care unit following acute isoniazid ingestion as a suicide attempt.

## 2. Case Report

History begins when the child had intentionally ingested, 40 isoniazid tablets, each of 10 mg amounting to a total dose of 4000 mg.

The tablets belonged to a parent currently taking the medication for active pulmonary tuberculosis.

The patient was admitted to the emergency ward four hours upon ingestion for tonic-clonic seizures which resolved after diazepam administration.

On examination, the patient was anicteric and eupneic, with a heart rate of 80 beats per minute, blood pressure of 12/7 cmHg, and SpO_2_ to 95% on room air.

On neurologic examination, the patient was postictal, with a GCS of 14/15 (E4V5M5).

The physical exam was otherwise unremarkable.

An initial workup made included head CT, complete blood count, full metabolic panel, liver function tests, creatine phosphokinase (CPK), prothrombin time, and a blood and urine toxicology screen. ABG drawn was normal with pH of 7.42,·HCO^3−^ of 25.9,·PaCO_2_ of 39.4, and normal electrolyte levels. Head CT was also normal. Initial lab values were Hb 10.9 g/dl, WBC 6270 elmts/mm^3^, and platelets 183 000 elmt/mm^3^. Liver enzymes were ALAT of 23 UI/l and ASAT of 17 UI/l. PT was 95%. CPK was 132 *µ*g/l (normal range: 0–150 *µ*g/l).

The patient did not present any new seizures after her admission and throughout her stay, but her liver function test values rised dramatically. CPK levels increased in a similar way, and her PT remained within normal range (Table 1).

## 3. Discussion

Isoniazid (INH), also known as isonicotinyl hydrazide, is an antituberculosis drug commonly prescribed in our country along with rifampicin, ethambutol, streptomycine, and pyrazinamide for the treatment of pulmonary and extrapulmonary tuberculosis (TB), as well as its common use for latent tuberculosis infection prophylaxis [1, 2].

INH has the following pharmacokinetic characteristics: INH is rapidly absorbed upon administration, though reduced when INH is taken with food. INH diffuses quickly to all body fluids and tissues and accumulates mostly in the liver. The volume of distribution (Vd) is 0.6 l/kg. It undergoes a first pass effect and has negligible protein binding. Time to peak blood concentration is within 1-2 hours following a single 300 mg oral dose. At the overdose situation, the peak plasma level of 3–7 mg/l is reached within 1.5–3 hours [3].

INH has structural similarity with pyridoxine (vitamin B6). Accumulation of INH causes functional deficiency of pyridoxine. As a result, metabolic functions dependent on pyridoxine are adversely affected. Neuropathic symptoms including seizures are caused due to deficient pyridoxine; this is one of the major adverse reactions with INH. Pyridoxine activity is decreased by INH, giving rise to clinical pyridoxine depletion. This occurs by two mechanisms: first, INH inhibition of enzyme pyridoxine phosphokinase, which converts pyridoxine to its active pyridoxal phosphate [3]; second, INH binding to pyridoxal phosphate, forming an inactive hydrazone complex that is excreted in the urine. Acidosis contributes to neuropathic symptoms due to the anion gap and increased accumulation of acidic metabolites of INH. Rhabdomyolysis, which is a complication of INH toxicity, is caused by direct damage to muscles by INH or its metabolites or as a consequence of seizures [4]. Agranulocytosis is probably an idiosyncratic adverse reaction to INH [5]. Psychosis and abnormal behavior have also been reported in children following its intake [6].

Metabolism of INH occurs by acetylation. Genetically, slow and fast acetylators are observed as two subgroups because of genetic polymorphism of the *N*-acetyltransferase-2 enzyme [7]. In both subgroups, hydrazine metabolites are responsible for hepatotoxicity, which should be suspected upon abnormal liver function tests, particularly ALAT [8].

INH doses of 35–40 mg/kg (8 tablets of 300 mg INH = 2400 mg) uniformly produce seizures. The acute ingestion of greater than 1.5 g leads to minor toxicity; whereas, ingestion of more than 6–10 g (i.e., 100–150 mg/kg, 20–30 tablets of 300 mg INH) is usually fatal without aggressive treatment [9].

INH poisoning in children is rare, and up to 1980, only 27 cases were reported [10]. A case of a toddler with INH poisoning presenting with general seizures was published as well [11]. Caksen et al. report the case of a two-year girl presenting with general seizures refractory to three antiepileptic drugs and only subsiding after pyridoxine administration [12]. Most case series encountered in the literature remain a bit outdated and very scarce when it comes to INH overdose in children.

The initial management of INH intoxication requires gastrointestinal decontamination with gastric lavage, preferably with activated charcoal [13], stabilization of vital signs with provision of patent airway and IV sodium bicarbonate should severe metabolic acidosis be present, cardiovascular support with IV fluids, and vasopressors. IV pyridoxine has been found to be highly effective for INH intoxication and should be administered to all symptomatic patients.

Parish and Brownstein suggested giving pyridoxine in a gram for gram basis to children with a high suspicion of isoniazid overdose, owing to history or previous ingestion, without awaiting laboratory results [14]. “It can be safely given at a rate of 5 g per 3 minutes in a 50 mL volume. In fact, serum INH determinations are not available in many emergency departments and have not been shown to correlate closely with symptomatology. They also noted that to potentiate the antidotal effects of pyridoxine, diazepam (0.1 mg/kg) can be given intravenously, preferably at a second intravenous site” [14].

Moreover, oral form of pyridoxine can be given should the intravenous presentations be unavailable [15].

INH can also be cleared through hemodialysis which should be begun the earliest possible due to its rapid absorption [16, 17].

Our patient presented to our emergency department four hours after ingesting 90 mg/kg of INH because of tonic-clonic seizures resolved upon diazepam administration.

Her stay was marked with deteriorating liver function tests and rhabdomyolysis without exhibiting symptoms of neither liver nor renal failure.

Toxic screening did not detect INH in serum or urine.

She remained symptom free during her stay, and her lab values were back within normal range the day of her discharge.

## 4. Conclusion

INH, yet uncommon, should not be overlooked especially in children with unexplained status epilepticus. Pyridoxine, its known antagonist, should be readily available in all emergency wards and intensive care units. Treatment remains symptomatic and includes organ support depending on the failing function.

## Figures and Tables

**Table 1:** Evolution of LFT and CPK during hospital stay.

	Admission	Day 2	Day 3	Day 4	Day 5
ALAT (UI/l)	23	37	89	263	319
ASAT (UI/l)	17	11	22	73	101
CPK (*µ*g/l)	132	2038	8450	35128	36934
PT (%)	95	95	93	92	
